# Dynamic risk prediction of survival in liver cirrhosis: A comparison of landmarking approaches

**DOI:** 10.1371/journal.pone.0306328

**Published:** 2024-07-05

**Authors:** Mitchell Paukner, Daniela P. Ladner, Lihui Zhao

**Affiliations:** 1 Department of Preventive Medicine, Feinberg School of Medicine, Northwestern University, Chicago, IL, United States of America; 2 Northwestern University Transplant Outcomes Research (NUTORC), Comprehensive Transplant Center (CTC), Feinberg School of Medicine, Northwestern University, Chicago, IL, United States of America; Cleveland Clinic, UNITED STATES

## Abstract

Electronic health records (EHR) data provides the researcher and physician with the opportunity to improve risk prediction by employing newer, more sophisticated modeling techniques. Rather than treating the impact of predictor variables on health trajectories as static, we explore the use of time-dependent variables in dynamically modeling time-to-event data through the use of landmarking (LM) data sets. We compare several different dynamic models presented in the literature that utilize LM data sets as the basis of their approach. These techniques include using pseudo-means, pseudo-survival probabilities, and the traditional Cox model. The models are primarily compared with their static counterparts using appropriate measures of model discrimination and calibration based on what summary measure is employed for the response variable.

## 1 Introduction

In modern medicine, the collection and storage of patient health data no longer restricts physicians or clinicians to using snapshot measurements in predicting health trajectories. Rather, the availability of comprehensive, longitudinal sequences of risk factors and biomarker changes has served as the impetus for innovation in risk prediction methodology. Leveraging this wealth of electronic health records (EHR) data has become a focal point in the medical and bio-informatics literature [1–4]. Simple statistical tools can be applied to these vast EHR data sets for meaningful analysis, but taking full advantage of the longitudinal nature of these data should lead to improved prediction of health outcomes in personalized settings. The practice of incorporating health histories through time-dependent covariates in modeling is known as dynamic risk prediction (DRP). There is substantial variation in the literature on DRP ranging from methods incorporating multi-linear sparse logistic regression [5], deep neural networks [6], landmarking [7], joint modeling [8], and many other statistical techniques.

The landmarking (LM) technique presents itself as desirable for its methodological simplicity and flexibility. The LM method has emerged as a popular option when attempting to dynamically predict outcomes in an EHR setting, updating specified risk prediction models at selected LM times [9, 10]. Some methods create a variable that measures the change in biomarker measurements from one LM time to the next [10], while others simply incorporate a time variable to interact with selected covariates across the different LM times [11]. While each method can be advocated for based on varying statistical or philosophical advantages, the underlying goal is leveraging longitudinal EHR data to improve the predictive accuracy of relevant health events for patients. This paper takes a simplified approach by incorporating an interaction between the selected covariate levels and a time variable.

Naturally, an equally important component of a DRP model along with covariates is the summary measures for the outcome. Several authors have used landmarking to dynamically model survival probability using a Cox Model [9, 10], while others have explored the pseudo-observation (PO) approach both through survival probability [12] and restricted mean survival time (RMST) [11]. We take a look at both survival probability and RMST and attempt to compare their relative value in an applied setting. With different outcome measures come different measures of prediction accuracy, making any direct comparison potentially misleading. Thus, direct comparison between dynamic methods is avoided, and we focus on comparing the performance of the dynamic models with their static counterparts for each summary measure.

The medical paradigm in which these DRP models will be assessed is the overall survival of patients with liver cirrhosis. Cirrhosis is a leading cause of mortality in the United States, implicated in the death of more than 50,000 patients each year, a number similar to diabetes and pneumonia [13–15] and exceeding most cancers [16, 17]. It remains imperative among hepatologists to identify new ways to maximize the wealth of longitudinal EHR data to improve the prediction of survival outcomes for cirrhotic patients.

The goal of this paper is to compare several different DRP modeling techniques based on the utilization of LM data sets. Specifically, we aim to compare the employment of PO or Cox regression for modeling survival outcomes for subjects diagnosed with liver cirrhosis. These DRP models can all utilize the LM framework but differ in terms of how the survival outcome is summarized (survival probability or RMST) as well as what types of models are employed (Cox model or generalized estimating equations). In Section 2 we will describe the model-building process for each method we intend to compare as well as the data used in training and testing our models. In Section 3 we explore the model performance based on various metrics such as C-statistics and Brier Scores. And, in Section 4 we discuss the various advantages and disadvantages of the modeling procedures.

## 2 Methods

The first stage in our modeling process is the construction of LM data sets to be used in model fitting. This process is relatively straightforward and begins with selecting a set of LM times *ℓ*_*j*_. We chose to select a set of 11 landmark times that take place every 0.5 years of follow-up (6 months) for our data analysis. Then, at each LM time *ℓ*_*j*_ we construct a data set *L*_*j*_ that excludes subjects who experienced their event or were censored prior to *ℓ*_*j*_.

Once the LM data sets are created they can be used in fitting various types of DRP models. Either the LM data sets *L*_*j*_ can be combined (“stacked”) and used in fitting a “super-model” [11], or a new model can be constructed at each *ℓ*_*j*_ [10]. Due to the issue of censoring, many of the standard modeling techniques used in dynamic risk prediction (DRP) are not tenable. In this paper, we will compare the use of PO with the standard use of a Cox proportional hazards model.

### 2.1 Pseudo observation approach

The basic concept behind the PO approach is to overcome the issue of censoring by creating a measurement of survival for every subject regardless of event or censoring status. This measurement is created through a transformation of the time-to-event data taking the following form:

$$\begin{array}{c} Z_{i}=nZ-\left(n-1\right)Z^{-i}\;\;\text{for}\;\;i=1,\ldots ,n \end{array}$$ (1)

where *Z* is the global survival outcome of interest and *Z*^−*i*^ is the outcome of interest computed while excluding the *i*^*th*^ subject. A PO of this form can be computed for all subjects and can roughly be interpreted as the subject-specific contribution to whatever survival metric is being used. Then, this measurement can be utilized as an outcome variable in a generalized linear model (GLM) [18].

A PO can, in theory, take several different forms, but in application, pseudo-means (PM), and pseudo-probabilities (PP) are intuitive and useful. Due to the challenges of estimating overall means in survival analysis in the presence of censoring, we will be using RMST and computing pseudo-RMST values as outcome variables. The term pseudo-mean will be used to indicate a pseudo-RMST value for the duration of the paper.

RMST, among various other mean-based statistics, has become a regular fixture in survival analysis when considering how to circumvent the issues of modeling and interpreting survival data when hazards are expected to be non-proportional (NPH) [19–23]. RMST can be computed and interpreted as the mean survival time from the beginning of follow-up to some time horizon *τ*. Thus, it estimates the expected survival duration of a patient until *τ*. This allows physicians and patients to consider survival outcomes in terms of days, months, and years of life gained or lost based on important covariates. The purpose for using RMST rather than overall mean survival is to account for the limited follow-up time due to censoring. In time-to-event analysis, many subjects won’t have their event time recorded if they are either lost to follow-up or the study ends prior to their event occurring. Thus, overall means cannot be directly estimated and may not be identifiable, though various restricted means can.

A restricted mean, $\mu \left(\tau \right)=\int_{0}^{\tau }S\left(t\right)dt$, is graphically represented as the area under the survival curve from randomization (*t* = 0) to some time horizon (*t* = *τ*). In practice, *μ*(*τ*) can easily be estimated by computing the area under the Kaplan-Meier (KM) curve between 0 and *τ* as follows:

$$\begin{array}{c} \hat{\mu }\left(\tau \right)=\sum_{t_{i}\leq \tau }\left(t_{i+1}^{*}-t_{i}\right)\hat{S}\left(t_{i}\right) \end{array}$$ (2)

where *t*_*i*_ is the *i*^*th*^ event time, $t_{i+1}^{*}=min\left(t_{i+1},\tau \right)$, and $\hat{S}\left(t\right)$ is the KM estimate of its respective survival function.

The subject-specific PMs to be used as the response variable *Y*_*i*_(*τ*) in a GLM or GEE, where *τ* is the restriction time, is computed as:

$$\begin{array}{c} {\hat{\mu }}_{i}\left(\tau \right)=n\hat{\mu }\left(\tau \right)-\left(n-1\right){\hat{\mu }}^{-i}\left(\tau \right)\;\;\text{for}\;\;i=1,\ldots ,n \end{array}$$ (3)

where $\hat{\mu }\left(\tau \right)$ is the restricted mean computed based on the KM curve with all subjects and ${\hat{\mu }}^{-i}\left(\tau \right)$ is the restricted mean computed based on the KM curve excluding subject *i* as in Eq 2. This particular technique was popularized by PK Andersen [24, 25], and has been utilized by several others in DRP modeling in survival analysis [11, 26, 27]. In our case, we compute the PM based on the conditional KM curve, where survival up to time *ℓ* is the condition. This PM can be interpreted as subject *i*’s individual contribution to the conditional RMST.

These PMs are then used as the outcome variable in a GEE in order to model the covariate effects on survival. Hence, we can model the effects of covariates on the PMs as:

$$\begin{array}{c} g\left[{\hat{\mu }}_{i}\left(\ell_{j},\tau |X_{i}\left(\ell_{j}\right)\right)\right]=\alpha_{j}+X_{i}{\left(\ell_{j}\right)}^{T}\beta \left(\ell_{j}\right) \end{array}$$ (4)

where *α*_*j*_ is the intercept, *β*(*ℓ*_*j*_) = (*β*_1_(*ℓ*_*j*_), *β*_2_(*ℓ*_*j*_), …, *β*_*p*_(*ℓ*_*j*_)) are the coefficients of the *p* predictor variables, **X**_**i**_(*ℓ*_**j**_) = (**X**_**i1**_(*ℓ*_**j**_), **X**_**i2**_(*ℓ*_**j**_), …, **X**_**ip**_(*ℓ*_**j**_)) are the values of the covariates collected until time *ℓ*_*j*_, and *g*() is a link function. Ultimately, we used the identity link function.

Interactions between covariates and *ℓ* were included in the models. For instance, the parameter *β*(*ℓ*) = (*β*_1_(*ℓ*), *β*_2_(*ℓ*), …, *β*_*p*_(*ℓ*)) could be a vector of functions that describe changes in the covariate effects according to variations of *β*_*p*_(*ℓ*) = *β*_*p*0_ + *β*_*p*1_*ℓ* + *β*_*p*2_*ℓ*^2^. If one were to apply some model selection criteria to model coefficients, the linear time interaction, or the squared time interaction might be excluded. While others who have used this approach to dynamically modeling EHR data have performed model selection through some type of step-wise technique, we will use a full model for the sake of comparability between our different modeling approaches. Functions from “geepack” in R were used for fitting the GEE.

When using a PP rather than a PM, one must modify Eq 1 to replace the RMST estimates $\hat{\mu }\left(\tau \right)$ and ${\hat{\mu }}^{-i}\left(\tau \right)$ with estimated survival probabilities $\hat{p}\left(\tau \right)$ and ${\hat{p}}^{-i}\left(\tau \right)$ at time *τ* based on the KM curve. The remaining modeling techniques will remain the same, though the interpretation of coefficients must be altered appropriately.

### 2.2 Cox proportional hazards model approach

The LM approach has been applied through the use of the well-known Cox model also. [9, 10] An obvious advantage to the Cox model is familiarity with statistical software packages, though its reliance on the proportional hazards assumption remains to be a relevant downside (especially when incorporating many predictor variables in modeling).

The dynamic Cox model can be expressed as:

$$\begin{array}{c} P\left(T_{i}>\tau |T_{i}>\ell_{j},X_{i}\left(\ell_{j}\right)\right)=\text{exp}\{-\Lambda_{0}\left(\tau |\ell_{j}\right)\text{exp}\left(\alpha^{\prime }X_{i}\left(\ell_{j}\right)\right)\} \end{array}$$ (5)

where Λ_0_(*τ* ∣ *ℓ*_*j*_) is the baseline cumulative hazard at time *τ* given survival to *ℓ*_*j*_ and *α*_*j*_ is a vector of regression parameters to be estimated during model fitting. Estimates of *α*_*j*_ can be obtained by maximizing the partial likelihood as would be done in the static case.

Either a new Cox model can be fit at each LM time with its corresponding LM data set, [10], or a “super-model” can be constructed using a stacked LM data set. [9] In this paper, we have chosen to use the method of stacking data sets based on the results of Keogh et al. in which the models fitted from stacked data sets had better performance.

### 2.3 Static models

In order to assess the value of each dynamic model, we also created several static models for comparison. The first, and most basic static model fits a baseline model with covariates available at time *t* = 0, and uses baseline covariates obtained at time *t* = 0 for survival prediction at time *t* = *ℓ* + *τ*, conditional on survival up to *ℓ*. Hence, if we set *τ* = 3, then at landmarking time *ℓ* = 3 we would be comparing the dynamic models’ 3 year survival predictions (using covariates at *t* = 3) against this static model’s 6-year survival predictions conditional on survival up to year 3 (using covariates at *t* = 0). This was the static model utilized by Yang et al. (2021) and will be referred to as SM1 [11].

The next static model constructed also fits a single model based on subjects’ baseline characteristics at time *t* = 0 but performs survival prediction based on subject covariates collected at time *t* = *ℓ*. Essentially, a baseline model is constructed, but updated covariates are used with this model in performing prediction at *t* = *τ*. Though this model uses updated patient information, it is not dynamic since it doesn’t take into account any element of patient history, only the patients’ current covariate values. This modeling approach will be referred to as SM2.

The final static model used for comparison fits a new prediction model at each landmark time (rather than only one at baseline) and also performs predictions based on the most recently available covariates at landmark time *ℓ*. This model may seem like it is encroaching on “dynamic” territory, but it should still be considered static as the model only accounts for covariates measured at a specific time point rather than a patient’s health history or progression of health indicators. This final modeling approach will be referred to as SM3.

### 2.4 Data

We used the Chicago Area Patient-Centered Outcomes Research Network (CAPriCORN) data which capture the diverse population of the greater Chicago metropolitan area. The dataset encompasses 30 hospitals, and 10 health systems, totaling 12.8 million patients, which allows us to model and assess mortality in patients with liver cirrhosis. The longitudinal nature of this EHR data allows us to compare various dynamic and static prediction models. The CAPriCORN data merges institutional databases while protecting patient health information. The CAPriCORN data was pulled for use by the authors on September 8, 2022, and was fully de-identified prior to any author accessing it.

There were numerous possible predictor variables available in the CAPriCORN data. We chose a subset of possible predictors based on relevant hepatological factors. The time-invariant variables utilized were basic demographic factors such as sex and age. The other important baseline variables are known as “cirrhosis etiology” which is a categorization of the cause of liver cirrhosis (e.g. alcohol use disorder or primary biliary cirrhosis). An individual can have multiple etiologies, so the three etiologies considered in this paper were coded as a 1 if a subject ever was diagnosed with that etiology and a 0 otherwise. The time-dependent covariates associated with liver health were the various relevant lab measurements of liver health (meld-sodium score and albumin level), and certain liver-related health events, known as decompensating events. Also, hepatocellular carcinoma was included as a time-dependent covariate.

The need for written informed consent and ethics approval was waived by the Northwestern University institutional review board due to the retrospective nature of the study. The data were de-identified by the National Patient-Centered Clinical Research Network prior to our use. All methods were carried out in accordance with relevant guidelines and regulations.

### 2.5 Model assessment

In order to assess the accuracy of the prediction models, we evaluate both discrimination and calibration. We measure discrimination in our models by using Harrell’s C-index as a form of determining how well each of our dynamic and static models assigns risk based on concordance [28]. This measure is computed as the area under the receiver operating characteristic curve and is thus commonly known as AUC. For the general case, AUC can be computed as:

$$\begin{array}{c} \;\text{AUC}\;=\frac{\sum_{i,j}1_{T_{j}<T_{i}}\cdot 1_{\eta_{j}>\eta_{i}}\cdot \delta_{j}}{\sum_{i,j}1_{T_{j}<T_{i}}\cdot \delta_{j}} \end{array}$$ (6)

where *η*_*i*_ indicates the risk score at the prediction time time *t* = *τ* that can be substituted for ${\hat{\mu }}_{i}$ in the RMST case or ${\hat{p}}_{i}$ in the survival probability case. The indicator functions, such as $1_{T_{j}<T_{i}}$, are 1 when the specified condition is met and 0 otherwise (*δ*_*j*_ is an event indicator). The AUC can range from 0 to 1 with scores closer to 1 indicating better prediction accuracy. Technically, scores near 0.5 indicate the worst prediction accuracy, as it is akin to flipping a coin.

When measuring calibration (the alignment between observed and predicted survival outcomes), we can take a uniform approach across the different modeling methods, but cannot necessarily make direct comparisons between them. In all cases, we use a Brier score or a slightly modified version of a Brier score for models using PM. Generally, the Brier score is used for measuring the accuracy of probabilistic predictions, such as with Cox models. The standard form for a Brier Score, in the absence of censoring, is as follows:

$$\begin{array}{c} BS=n^{-1}\sum_{i=1}^{n}{\left({\hat{p}}_{i}-1_{T_{i}>t}\right)}^{2} \end{array}$$ (7)

where $1_{T_{i}>t}$ is an indicator function that is 1 when the observed time for subject *i* is greater than the prediction time *t*. The Brier Score ranges from 0 to 1 with scores closer to 0 indicating better prediction accuracy.

We use a version of a Brier score that incorporates inverse probability censoring weights (IPCW) in order to accommodate for the censoring in our data. Thus, we compute the Brier Score as:

$$\begin{array}{c} BS\left(t\right)=\frac{1}{N}\sum_{i=1}^{N}(\frac{{\left(0-{\hat{p}}_{i}\right)}^{2}\cdot 1_{T_{i}\leq t,\delta_{i}=1}}{\hat{G}(T_{i}^{-})}+\frac{{\left(1-{\hat{p}}_{i}\right)}^{2}\cdot 1_{T_{i}>t}}{\hat{G}\left(t\right)}) \end{array}$$ (8)

where $\hat{G}(t)$ is the estimator of the conditional survival function of the censoring times calculated using the KM method, $1_{T_{i}\leq t,\delta_{i}=1}$ is an indicator function that is 1 when the observed time for subject *i* is less than or equal to the prediction time *t* but is an event time, and $1_{T_{i}>t}$ is an indicator function that is 1 when the observed time for subject *i* is greater than the prediction time *t*.

In the PM case, since survival probabilities are not being estimated, we have the following form for our “Brier Score”:

$$\begin{array}{c} BS\left(t\right)=\frac{1}{N}\sum_{i=1}^{N}(\frac{{\left(T_{i}-{\hat{\mu }}_{i}\right)}^{2}\cdot 1_{T_{i}\leq t,\delta_{i}=1}}{\hat{G}(T_{i}^{-})}+\frac{{\left(t-{\hat{\mu }}_{i}\right)}^{2}\cdot 1_{T_{i}>t}}{\hat{G}\left(t\right)}) \end{array}$$ (9)

This resulting “Brier Score” does not have an upper bound of 1, and is not directly comparable to any Brier Score computed based on survival probability, though lower scores do indicate better performance.

For model assessment, we utilized 75% of the available data set for training our model and the remaining 25% for model assessment.

## 3 Results

The size of the entire CAPriCORN data set available for model building was *n* = 7040. In the overall cohort (*n* = 7040), there is a median follow-up time of 1.12 years ranging from 0.0027 to 11.01 years to death or loss-to-follow-up. During the follow-up period, 1371 subjects (19.47%) died. The overall 3-year survival rate was 28.13%, and the 8-year survival rate was 3.98%. Dynamic and static models were constructed with a *τ* = 3 years with landmark times of *ℓ* = 0, 0.5, …, 4.5, 5 years. Table 1 displays the coefficients with their standard errors and p-values included in the dynamic models, and Table 2 displays the results from the SM2 static model fit using baseline covariates for the sake of comparison.

**Table 1:** The results of the dynamic models with *τ* = 3 and mortality as the event of interest.

Variable	Time Function	PM			PP			Cox Model		
		$\hat{\beta }$	SE	p-value	$\hat{\beta }$	SE	p-value	$\hat{\beta }$	SE	p-value
**Intercept**	1	2.833	0.047	< 0.0001	0.863	0.026	< 0.0001			
**Age**	1	-0.009	0.001	< 0.0001	-0.004	0.001	< 0.0001	0.037	0.002	< 0.0001
	*ℓ*/5	0.025	0.004	< 0.0001	0.009	0.002	0.0001	-0.085	0.015	< 0.0001
	(*ℓ*/5)^2^	-0.021	0.005	< 0.0001	-0.008	0.003	0.0018	0.056	0.018	0.0398
**ETOH**	1	0.171	0.025	< 0.0001	0.055	0.012	< 0.0001	-0.317	0.068	0.0021
	*ℓ*/5	-0.753	0.127	< 0.0001	-0.278	0.068	< 0.0001	1.677	0.466	0.0169
	(*ℓ*/5)^2^	0.554	0.131	< 0.0001	0.183	0.073	0.0124	-1.166	0.546	0.1376
**Biliary**	1	0.066	0.052	0.2025	0.006	0.029	0.8487	-0.223	0.152	0.2686
	*ℓ*/5	-0.710	0.252	0.0049	-0.312	0.148	0.0353	3.344	0.927	0.0055
	(*ℓ*/5)^2^	0.713	0.247	0.0040	0.366	0.150	0.0147	-3.601	1.070	0.0102
**Chol**	1	0.133	0.065	0.0391	0.069	0.034	0.0403	-0.412	0.214	0.1427
	*ℓ*/5	-0.094	0.310	0.7615	0.004	0.176	0.9826	-0.886	1.361	0.5862
	(*ℓ*/5)^2^	-0.105	0.315	0.7404	-0.162	0.186	0.3837	1.603	1.511	0.3767
**MELD-NA**	1	-0.026	0.002	< 0.0001	-0.009	0.001	< 0.0001	0.067	0.004	< 0.0001
	*ℓ*/5	0.064	0.010	< 0.0001	0.016	0.005	0.0014	-0.113	0.030	0.0113
	(*ℓ*/5)^2^	-0.049	0.011	< 0.0001	-0.009	0.006	0.1394	0.039	0.037	0.4795
**Albumin**	1	0.197	0.013	< 0.0001	0.089	0.006	< 0.0001	-0.565	0.043	< 0.0001
	*ℓ*/5	-0.452	0.059	< 0.0001	-0.140	0.030	< 0.0001	0.215	0.249	0.5015
	(*ℓ*/5)^2^	0.367	0.063	< 0.0001	0.117	0.033	0.0004	-0.413	0.286	0.2767
**Decomp**	1	-0.151	0.024	< 0.0001	-0.053	0.011	< 0.0001	0.230	0.041	0.0005
	*ℓ*/5	0.640	0.140	< 0.0001	0.215	0.073	0.0030	-0.828	0.416	0.1613
	(*ℓ*/5)^2^	-0.671	0.161	< 0.0001	-0.257	0.088	0.0036	1.095	0.519	0.1014
**HCC**	1	-0.205	0.067	0.0022	-0.087	0.030	0.0044	0.504	0.130	0.0265
	*ℓ*/5	-0.376	0.440	0.3924	-0.341	0.230	0.1379	2.956	0.925	0.0497
	(*ℓ*/5)^2^	0.200	0.507	0.6933	0.272	0.287	0.3424	-2.431	1.106	0.1296

**Table 2:** The results of the static models fit at baseline with *τ* = 3 and mortality as the event of interest.

Variable	PM			PP			Cox Model		
	$\hat{\beta }$	SE	p-value	$\hat{\beta }$	SE	p-value	$\hat{\beta }$	SE	p-value
**Intercept**	2.898	0.130	< 0.0001	0.896	0.061	< 0.0001			
**Age**	-0.011	0.001	< 0.0001	-0.005	0.001	< 0.0001	0.030	0.003	< 0.0001
**ETOH**	0.192	0.033	< 0.0001	0.054	0.016	0.0005	-0.397	0.077	< 0.0001
**Biliary**	0.085	0.069	0.2188	0.013	0.038	0.7340	-0.229	0.176	0.1936
**Chol**	0.179	0.088	0.0409	0.079	0.044	0.0768	0.490	0.244	0.0448
**MELD-NA**	-0.028	0.002	< 0.0001	-0.009	0.001	< 0.0001	0.058	0.004	< 0.0001
**Albumin**	0.215	0.023	< 0.0001	0.094	0.010	< 0.0001	-0.640	0.053	< 0.0001
**Decomp**	-0.131	0.028	< 0.0001	-0.048	0.012	0.0001	0.182	0.044	< 0.0001
**HCC**	-0.210	0.078	0.0073	-0.081	0.034	0.0188	0.483	0.141	0.0006

From Table 1 it is clear that the time-varying measures of MELD-Sodium score, Albumin level, and the number of decompensating events are highly statistically significant in the dynamic model, while the baseline covariates of age and etiology of alcoholic cirrhosis (ETOH) are also highly significant. From Table 2 we see that the same predictor variables are significant.

The time function used in this modeling procedure fits a linear and a quadratic interaction effect between time and the eight covariates of interest. It adds flexibility for modeling the effects of the covariates. A significant p-value with any of the interaction effects indicates a significant relationship between the specific covariate and time (either linear, quadratic, or both). More complicated interactions with time are possible (e.g., splines), though they extend outside the scope of this paper.

The dynamic coefficient of any covariate used in this modeling procedure can be calculated by the following formula:

$$\begin{array}{c} \beta_{p}\left(\ell_{j}\right)=\beta_{p0}+\beta_{p1}*\left(\ell_{j}/5\right)+\beta_{p2}*{\left(\ell_{j}/5\right)}^{2},\ell_{j}\in \left[0,5\right] \end{array}$$ (10)

For example, in the PM model at LM time *ℓ* = 5, the covariate effect of having a decompensating event is −0.151 + 0.64 * 1 − 0.671 * 1 = −0.182. So, holding all other variables constant, patients who experience decompensated at the LM time of 5 years are expected to lose 0.182 mean survival years compared to patients who don’t experience a decompensating event.

In practice, model selection criteria should be employed to potentially eliminate certain variables, including certain interaction effects. For example, it may make sense to exclude the linear and quadratic interaction effect between time and HCC status in the PM model as the p-values for those coefficients are high. For our purposes, we wanted each of the dynamic models to be as directly comparable as possible. Hence, we included all covariates and interactions in the final models.

**Fig 1:**
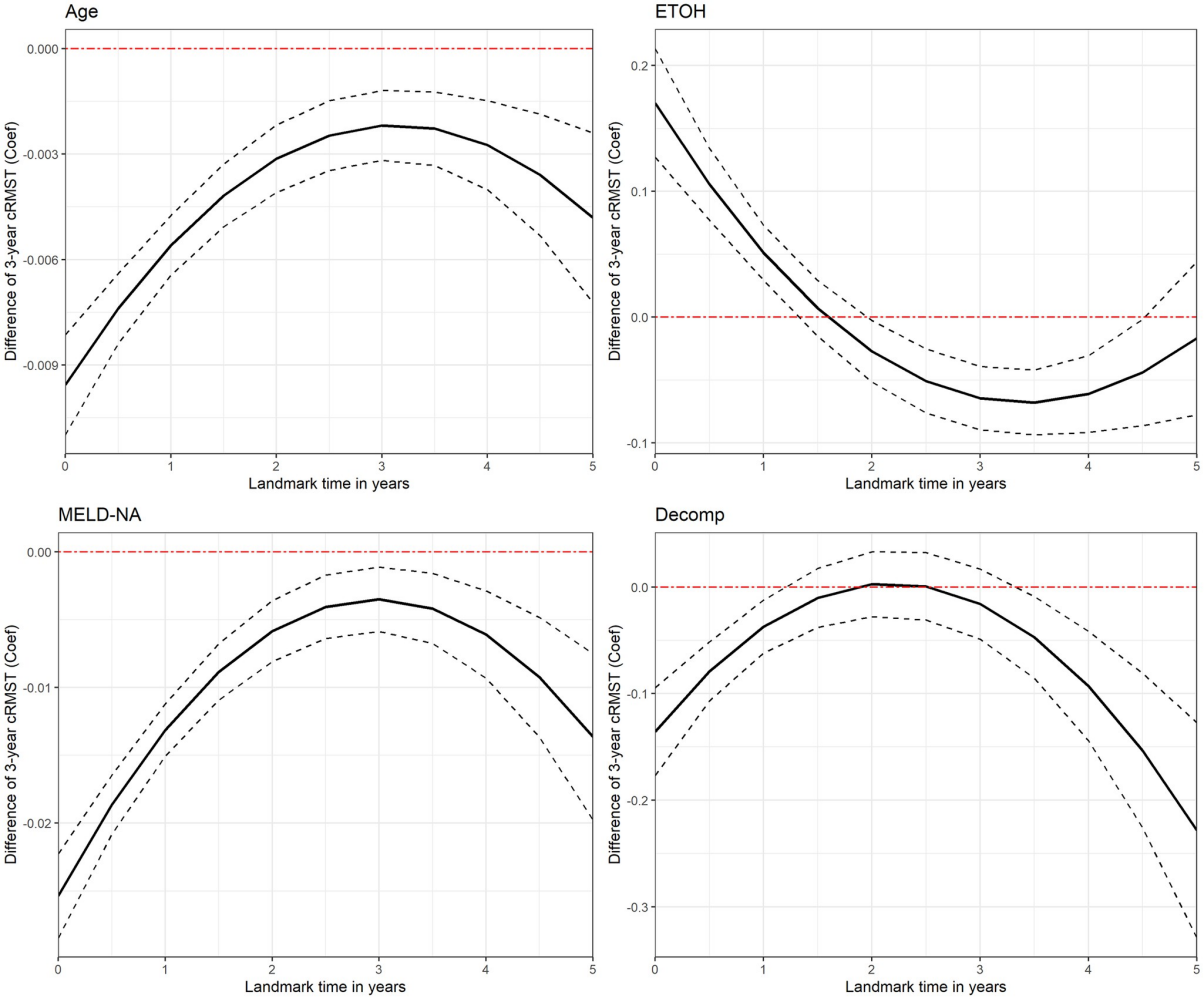
Difference in 3-year conditional RMST in the dynamic RMST model over the 5-year LM period. The solid line represents the dynamic coefficients *β*_*p*_(*ℓ*), or the difference in conditional 3-year RMST resulting from a single unit increase in the *p*^*th*^ covariate at *ℓ*. The black dashed line represents the 95% CI.

Fig 1 can be used to visualize the difference in 3-year conditional RMST dynamic coefficients over the 5-year LM period with 95% confidence intervals, while the same figures for the PP and Cox models can be found in the S1 Appendix. Essentially, at any specific LM (represented on the x-axis) one can see the covariate-specific effect (on the y-axis) of 3-year RMST of a one-unit increase in the variable of interest (while holding all other variables constant). Due to the inclusion of a quadratic time interaction in our model, the resulting 3-year conditional RMST dynamic coefficients will have a parabolic structure, so long as the quadratic term is not equal to 0. If we had only included a linear time interaction, we would instead see a straight line with some non-zero slope (as long as the interaction was non-zero), and had we included no time interaction they would be horizontal lines. The clinicians and physicians using the dynamic model with time interactions could then interpret the relationship between the landmarking times and the dynamic coefficients and better understand the relationship between time and the covariate-specific effect on survival.

### 3.1 Individual dynamic prediction

**Fig 2:**
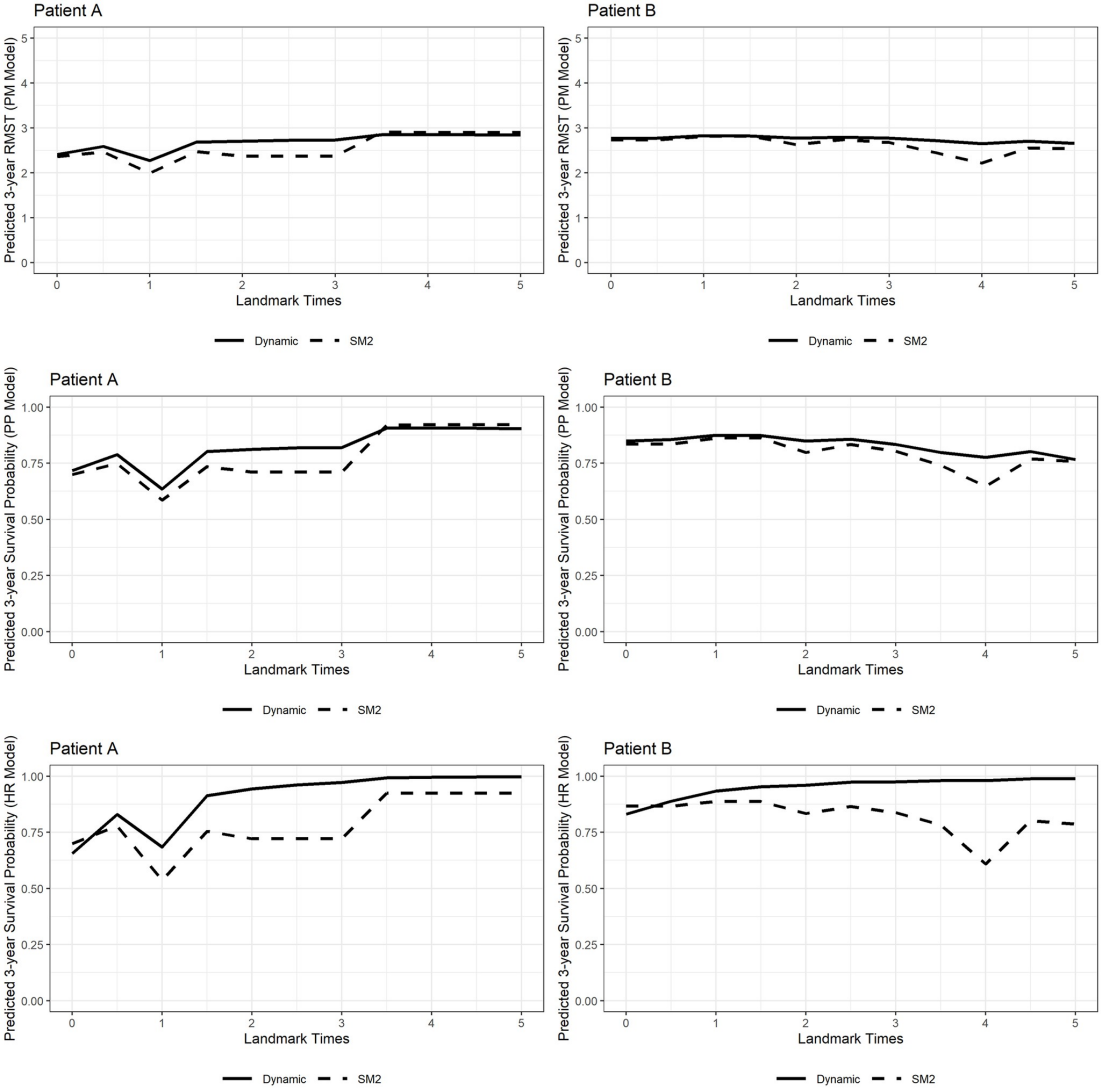
Individual predictions with the dynamic and static models for patients A and B with all-cause mortality as the event of interest. The solid line represents the dynamic 3-year survival prediction, and the dashed line represents the 3-year prediction based on the static model fit with baseline covariates at *t* = 0 (SM2).

We can also utilize the dynamic model to provide individual 3-year survival predictions for patients with liver cirrhosis. Fig 2 shows the individual predictions with the dynamic and static models for two different patients based on each model type. The solid line represents the dynamic conditional 3-year survival prediction, and the dashed line represents the 3-year survival prediction based on the static model fit with baseline covariates at the beginning of the study (SM2). In a clinical setting, it can be valuable to distinguish the risk of death between patients when evaluating candidates for certain treatment options (such as transplants). In Fig 2, patient A had a relatively high meld score through the duration of follow-up, experienced multiple decompensating events, and was diagnosed with hepatocellular carcinoma (HCC) at year 1, whereas patient B had a relatively low meld score until roughly year 4, experienced no decompensating events, and was never diagnosed with HCC. Notably, patient A experiences a large decrease in their predicted 3-year survival from *ℓ* = 0.5 to *ℓ* = 1 (dropping from 2.58 years to 2.27 years and from 2.46 years to 1.99 years in the dynamic and static RMST models respectively) when they were diagnosed with HCC. We also see that the dynamic models are not as strongly influenced by risk factors that take place at a specific time point. For example, when patient B experiences an increase in meld score around year 4, the static model predicts a sharper decrease in survival (2.21 years in the RMST model, 64.80% in the PP model, and 60.95% in the HR model) than does the dynamic model (2.64 years in the RMST model, 77.55% in the PP model, and 97.99% in the HR model) which incorporates the entire history of meld scores. In the event a clinician wishes to evaluate different candidates for transplant at different time points, comparing risk prediction could serve as a useful tool.

### 3.2 Model performance

**Fig 3:**
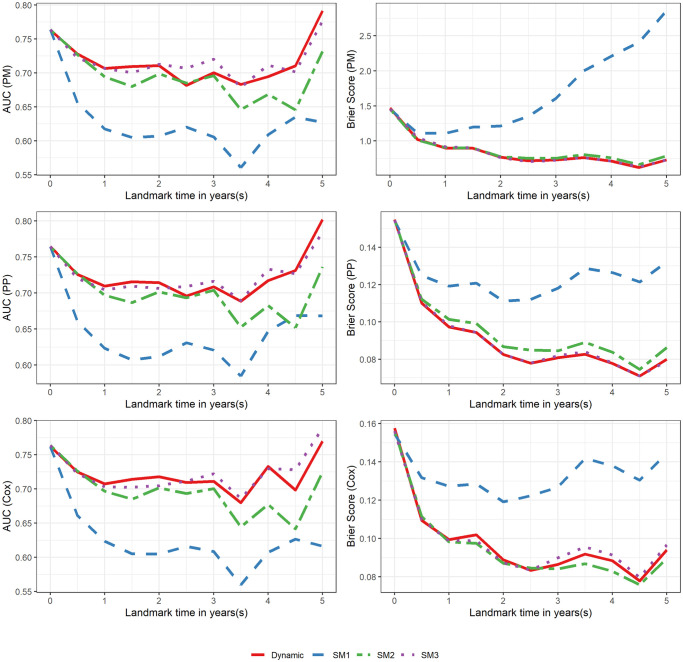
Assessment of model performance in terms of AUC and Brier score. The solid line represents the dynamic model and the dotted/dashed lines represent the 3 different static models.

As described in Section 2.5, the quality of each dynamic model’s performance was measured in terms of discrimination (AUC) and calibration (prediction error or Brier score) and compared with their various static counterparts. A higher AUC score indicates better model performance, while a lower prediction error or Brier score represents better model performance. Fig 3 shows the results of this comparison. It is evident, in terms of AUC, that the dynamic models outperform the crudest static model (SM1), but the static models that merely use updated covariate levels for predictions have similar performance.

When considering the calibration of these models, no static model appears to perform better across all 3 modeling procedures than their dynamic counterpart. In the PM model, the crude static model has a better prediction error than either of the other static models but has the worst Brier score in the PP model. Likewise, SM2 has the worst prediction error in the PM model, but the best Brier score in the Cox model. Essentially, even though the dynamic model is not uniformly better than its static counterparts in any given model, it has a very similar AUC and prediction error/Brier score to the best static models.

The strong performance of SM2 and SM3 might be due to their use of the most current covariate information when predicting outcomes. In many health settings, it may be the case that the most current health information does most of the leg work in terms of predicting future outcomes, and that any modeling procedure that incorporates this information will perform relatively well.

The dynamic model seems to be most advantageous when using the PM approach, and least advantageous when applying a Cox modeling procedure. Thus, determining what type of summary measure is desired when modeling should influence a researcher’s choice of modeling technique (dynamic or static) in a setting similar to ours. If a summary measure is desired in terms of survival duration, then a dynamic PM model might be best. Whereas, if the prediction is desired in terms of survival probability, and a standard Cox modeling procedure is desired, then a static model fit at baseline (such as SM2) might be a reasonable choice.

From this figure, we can see that SM2 and SM3 perform well in relation to the dynamic models. This may indicate that the most current health information may be of the greatest value when performing prediction.

## 4 Discussion

Dynamic risk prediction modeling is a vast research-scape and offers medical researchers a new paradigm for dealing with EHR data. In this paper, we have specifically touched on three different modeling techniques that apply the LM approach, but this accounts for a mere fraction of the various complex methods presented in recent literature.

Overall, the LM method proves to be intuitive, straightforward, and useful. Even though the dynamic models built from LM data sets were not an overwhelming favorite in terms of model performance, they were always at least as good as their static counterparts, and better in several specific cases.

One notable downside to the LM approach is the computational time involved in constructing the LM data set. The vast majority of computing time was devoted to this facet of modeling where the cutLM() function from the “dynpred” package in R was used. For smaller data sets, this step would not be too intensive, but with EHR data sets containing numerous subjects, this could become a process that requires extremely powerful computing equipment. On the other hand, simple static models could be rapidly built with little resources required.

Alternatively, a modeling procedure based on LM data sets could be employed in a similar fashion to Parast et al. where differences in the time-varying covariates are measured across the LM times and used as a predictor variable in model fitting. This is intuitive, however, Parast et al. found only marginal improvements in model calibration and no improvement in discrimination (as compared to a static model) while applying this methodology to Diabetes Prevention Program (DPP) data [10].

Since the CAPriCORN data is not available to the public, readers could perform our analysis using the “pbc2” data in the “dynpred” package in R. This data similarly measures survival in cirrhotic subjects and has been used to test the quality of DRP methods such as those presented by Yang et al. [11].

## 5 Conclusion

The ultimate goal of dynamic risk prediction is to improve the accuracy of personalized prediction rules for individual patients. As data collection becomes easier, faster, more expansive, and more accurate, this goal can become more attainable. While the quality of the predictions from the DRP models discussed in this paper may not be overwhelming, their use offers similar performance to static models and encourages the continued advancement in statistical methodology. Given the prevalent use of risk prediction modeling in medical settings, even simple advances in prediction quality can benefit many patients in the future.

## Supporting information

S1 Appendix (PDF)
