# Supplementary material for: Dynamic risk prediction of survival in liver cirrhosis: A comparison of landmarking approaches
Source: PLoS One. 2024 Jul 5;19(7):e0306328. doi: 10.1371/journal.pone.0306328 (PMC11226049; doi:10.1371/journal.pone.0306328)
Supplement: S1 Appendix — (PDF) [file pone.0306328.s001.pdf]

Dynamic Risk Prediction of Survival in Liver Cirrhosis: A Comparison of Landmarking Approaches: Supplemental Document

This document serves as a supplementary appendix to the manuscript "Dynamic Risk Prediction of Survival in Liver Cirrhosis: A Comparison of Landmarking Approaches". The figures were deemed important by the authors or the reviewers but did not fit into the main body of the paper.

1. SUPPLEMENTAL FIGURES

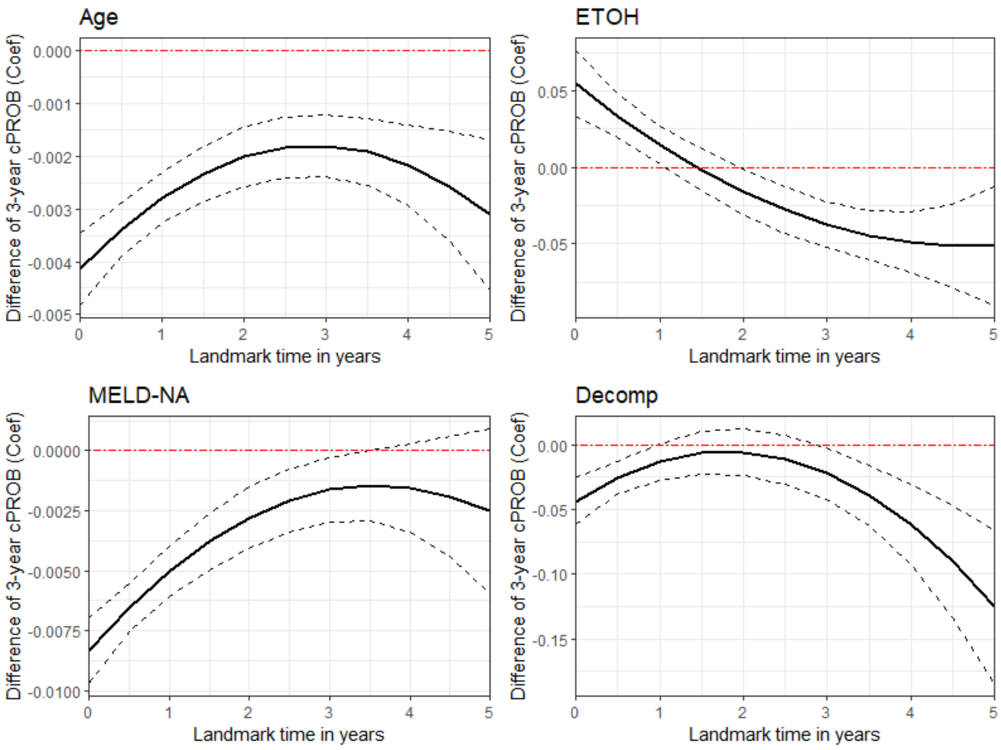

Fig. S1. Difference in 3-year conditional survival probability in the dynamic PP model over the 5-year LM period. The solid line represents the dynamic coefficients $\beta_p(\ell)$, or the difference in conditional 3-year survival probability resulting from a single unit increase in the pth covariate at ℓ. The black dashed line represents the 95% CI.

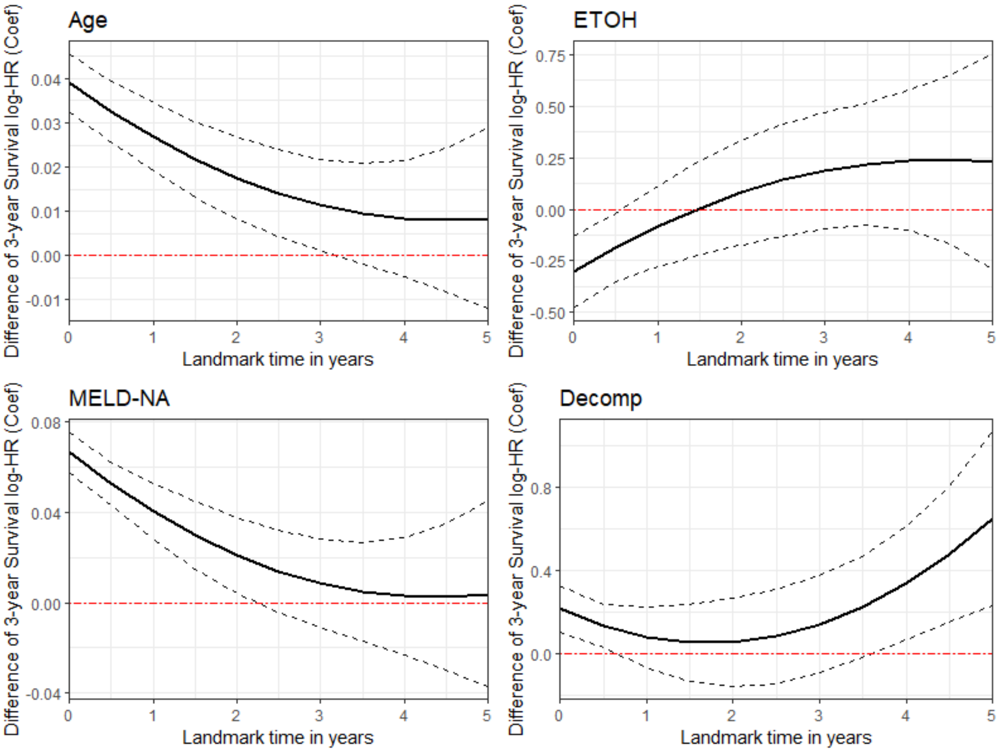

Fig. S2. Difference in 3-year conditional log-HR in the dynamic Cox model over the 5-year LM period. The solid line represents the dynamic coefficients $\beta_p(\ell)$, or the difference in conditional 3-year log-HR resulting from a single unit increase in the pth covariate at ℓ. The black dashed line represents the 95% CI.
